# A Rare Case of Hypercortisolemia Alongside Anorexia Nervosa

**DOI:** 10.7759/cureus.91225

**Published:** 2025-08-29

**Authors:** Zehra Kara, Murat Alay, Muhammed İ Erbay, Serhat Uysal, Pinar Kadıoglu

**Affiliations:** 1 Endocrinology, Diabetes and Metabolism, Istanbul University-Cerrahpaşa, Cerrahpaşa Medical Faculty, İstanbul, TUR; 2 Endocrinology, Diabetes and Metabolism, Van Yuzuncu Yıl University, Faculty of Medicine, Van, TUR; 3 Medicine, Istanbul University-Cerrahpaşa, Cerrahpaşa School of Medicine, Istanbul, TUR; 4 Endocrinology, Diabetes and Metabolism, Istanbul University-Cerrahpaşa, Cerrahpaşa Medical Faculty, Istanbul, TUR

**Keywords:** anorexia nervosa, excessive functionality, hypercortisolism, hypothalamic-pituitary-adrenal axis, pseudo-cushing's syndrome

## Abstract

We present a case of anorexia nervosa (AN), which is a rare cause of hypercortisolism. We discuss the case of a female patient with a body mass index (BMI) of 12.4 kg/m^2^ who was referred to us because of hypercortisolism. When the patient was evaluated for Cushing's syndrome (CS), the clinical presentation was not suggestive. However, hypercortisolism indicators showed CS. Pituitary and ectopic focus were not found in the radiological evaluation. The patient, who was noted not eating due to the fear of gaining weight, was consulted with a psychiatrist; a diagnosis of anorexia nervosa was made. Hypercortisolism is caused by conditions able to chronically activate the hypothalamic-pituitary-adrenal axis (HPAa), and although rare, it can also occur in psychiatric disorders such as AN. In patients diagnosed with AN, CS should be suspected if the typical clinical features of Cushing's syndrome are present. In AN-associated pseudo-Cushing's syndrome (PCS), an improvement in clinical features and laboratory parameters can be expected with appropriate weight gain. Recognizing PCS at presentation protects patients from unnecessary testing and treatment.

## Introduction

The Diagnostic and Statistical Manual of Mental Disorders, 5th Edition (DSM-5) diagnostic criteria for anorexia nervosa (AN) are known for their behavioral and psychological features, including restriction of food intake leading to low body weight, intense fear of gaining weight or becoming fat, and distortion of body image [1]. It is most common in adolescence. Every organ system can be affected due to weight loss [2]. We may encounter various complications related to malnutrition [2]. Endocrine glands and axes are also affected. Leptin, free triiodothyronine (fT3), and insulin-like growth factor-1 (IGF-1) decrease, while growth hormone (GH) and cortisol increase [3]. Anorexia nervosa has the highest mortality rate among psychiatric diseases [3]. Patients with AN are treated with nutritional rehabilitation, cognitive-behavioral therapy, and family therapy [3]. Anorexia nervosa is difficult to treat due to reasons such as denial, resistance to treatment, and discontinuation of treatment [3].

Anorexia nervosa is one of the rare causes of pseudo-Cushing's syndrome (PCS) [4]. In PCS, increased secretion of adrenocorticotropic hormone (ACTH) is seen without a tumor source [4]. Excessive functionality of the hypothalamic-pituitary-adrenal axis (HPAa) and corticotropin-releasing hormone (CRH) and/or arginine vasopressin (AVP) pathways causes secondary hypercortisolism causes pseudo-Cushing's symptoms [5]. Decreased cortisol clearance, changes in cortisol's affinity for glucocorticoid-binding globulin, and glucocorticoid receptor resistance are other mechanisms responsible for hypercortisolism in patients with AN [6]. Buffalo hump, hirsutism, and moon face are some of the symptoms in PCS [7,8]. The low weight of patients with AN and the absence of clinical symptoms can be attributed to glucocorticoid resistance [7,8].

Our case report mentions a patient who first came to our outpatient clinics and was shown to have hypercortisolism alongside anorexia nervosa that arose in the last one year.

## Case presentation

A 22-year-old woman contacted the endocrinology department of another university hospital complaining of amenorrhea, fatigue, and loss of appetite for the last year. In her medical history, she had no known history of disease or drug use. She was a nonsmoker and did not drink alcohol. In her family history, she had an aunt and a cousin who had lung cancer and an uncle who had breast cancer. There were no psychiatric illnesses in the family.

**Figure 1:**
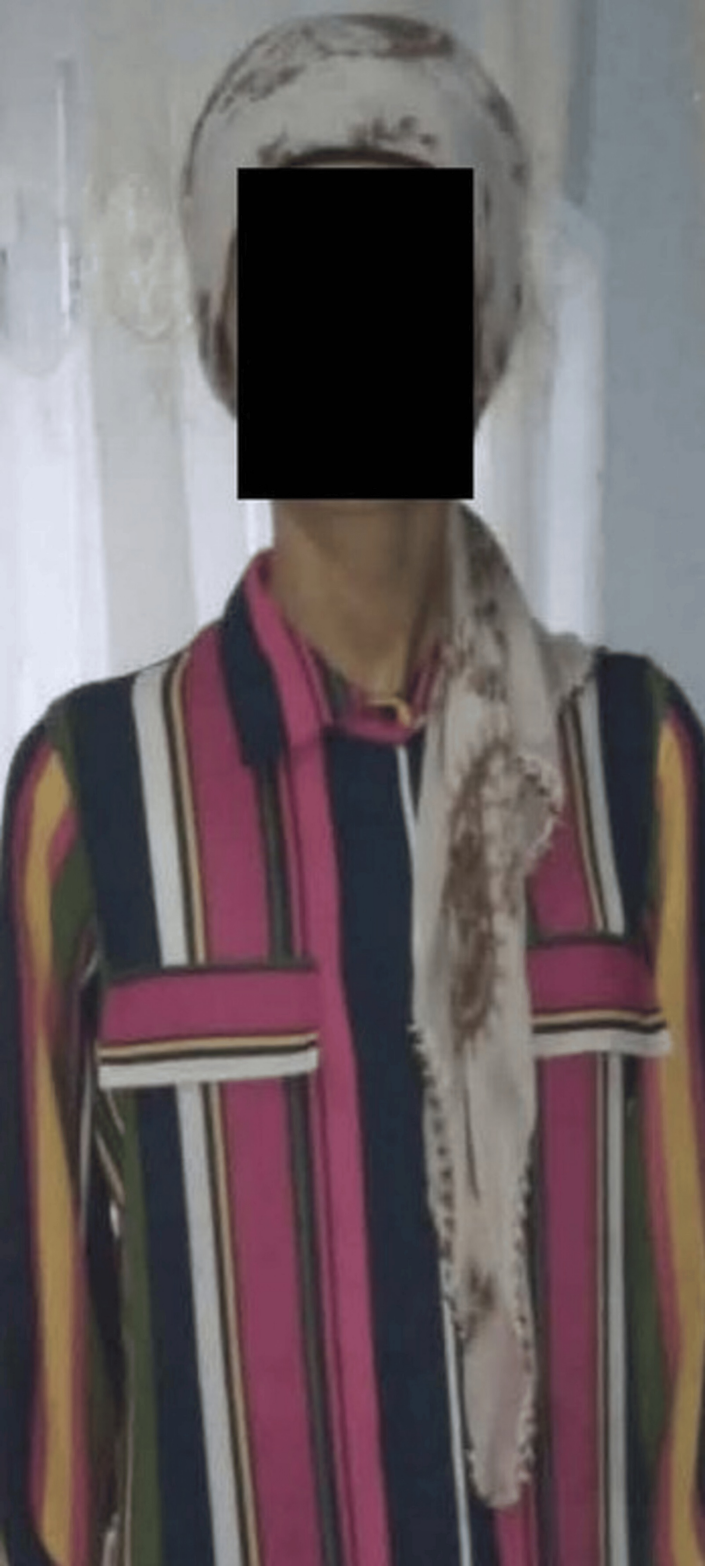
Patient's image taken in August 2022

The patient's cortisol axis was examined due to amenorrhea; the cortisol level was not suppressed by the dynamic tests. No pathology was detected in the blood count, glucose, kidney, and liver function tests. There was no electrolyte imbalance. Thoracic-abdominal computed tomography (CT) revealed no abnormality. Gallium-68 scintigraphy was within normal limits. The patient was referred to our tertiary pituitary center for sampling of the inferior petrosal sinus. The patient reported that she had lost 40 kg in one year. The physical examination revealed a body mass index (BMI) of 12.4 kg/m^2^ (Figure 1) and bilateral breast atrophy. The patient's laboratory results on admission to our clinic are shown in Table 1. Due to the dyspeptic symptoms and weight loss, an upper gastrointestinal endoscopy and colonoscopy were performed, which revealed antral gastritis. The ultrasound examination of the thyroid gland was normal. Body fat percentage measured with Tanita was 1%. Obstetric and gynecological examination was normal. Contrast-enhanced magnetic resonance imaging (MRI) of the pituitary gland revealed no pathology. Echocardiography showed mild to moderate mitral and tricuspid regurgitation, and a moderate (16 mm) pericardial effusion. The results of bone densitometry by dual-energy X-ray absorptiometry (DXA) were normal (Table 1).

**Table 1:** Laboratory values at admission and at six-month follow-up *Measured using the insulin tolerance test **From 24-hour urine 5-HIAA 5-HIAA: 5-hydroxy indoleacetic acid, DST: dexamethasone suppression test, ACTH: adrenocorticotropic hormone, FSH: follicle-stimulating hormone, LH: luteinizing hormone, DHEA-SO4: dehydroepiandrosterone sulfate, TSH: thyroid-stimulating hormone, fT3: free triiodothyronine, fT4: free thyroxine, IgA: immunoglobulin A, IgG: immunoglobulin G, HOMA-IR: Homeostatic Model Assessment for Insulin Resistance, DXA: dual-energy X-ray absorptiometry

Parameters	At admission	At 6-month follow-up	Reference range
Cortisol (µg/dL)	26	12	10-20
ACTH (pg/mL)	28	14	10-20
DST 1 mg (µg/dL)	20	0.5	<1.8
DST 2 mg (µg/dL)	18	-	<1.8
24-hour urine cortisol	2 times	Within the normal range	30-160
Nighttime salivary cortisol	1.5 times	Within the normal range	0.5-2
DST 8 mg (µg/dL)	15	-	50% suppression compared to baseline cortisol
Estradiol (pg/mL)	6	17	30-200
FSH (mIU/mL)	0.7	7	5-20
LH (mIU/mL)	0.3	4	5-20
17-OH progesterone (ng/mL)	1.3	-	<2
DHEA-SO4 (ng/mL)	135	-	65-368
Total testosterone (ng/dL)	37	-	6-82
TSH (mIU/L)	0.8	1.3	0.27-4.2
fT4 (ng/dL)	0.8	1.1	0.93-1.7
fT3 (ng/L)	0.9	-	2-4.4
Somatomedin C (mg/L)	39	-	107-367
Growth hormone (ug/L)	6.4	-	0.1-9.8
Growth hormone (mg/L)*	7.2, 7.8, 9.9	-	3
Prolactin (ng/mL)	12	-	10-25
5-HIAA (mg/day)**	4.9	-	2-9
Calcitonin (pg/dL)	1.2	-	<10
Anti-tissue transglutaminase IgA (U/mL)	0.08	-	<12
Anti-tissue transglutaminase IgG (A/mL)	0.25	-	<12
HOMA-IR	1.4	-	<2.5
DXA (Z scores)			
Femoral neck	0.4	-	>-2
Total lumbar	0.3	-	>-2

**Figure 2:**
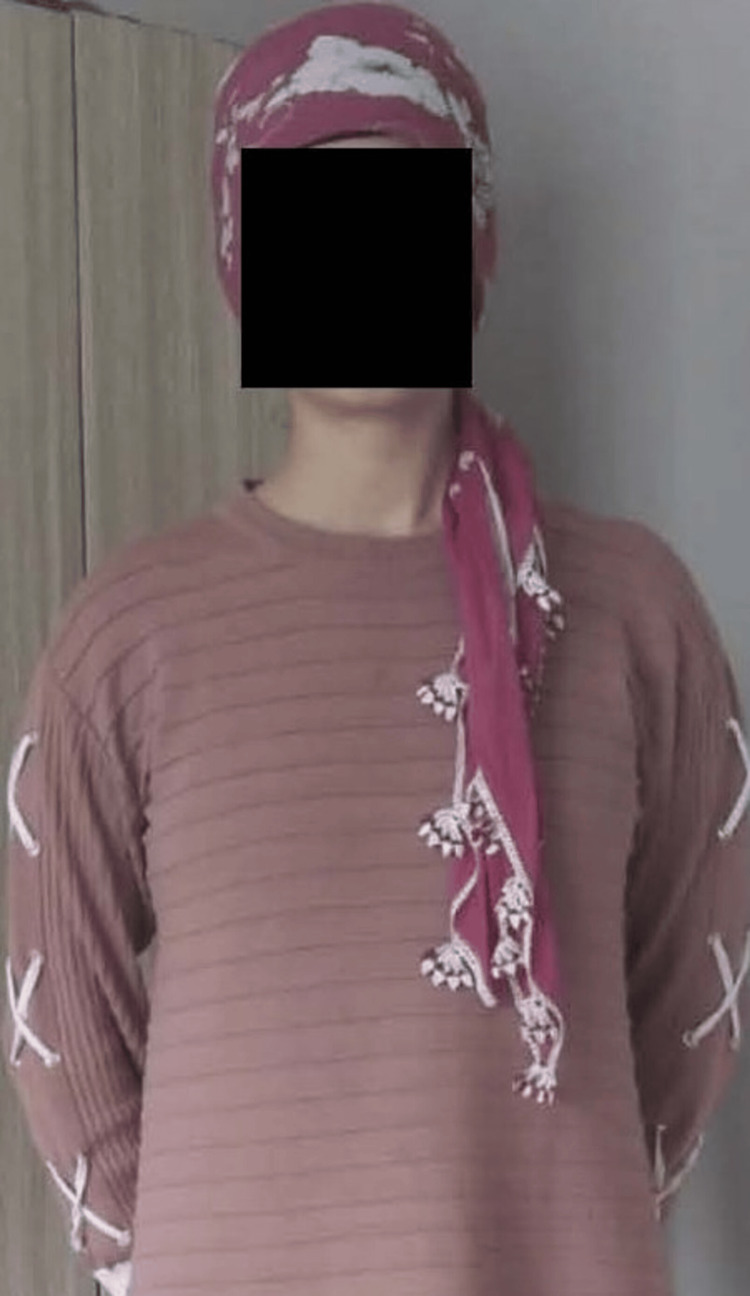
Patient's image taken in February 2023

The nutritional profile was drawn up by a dietitian. During the follow-up of the patient, it was observed that she refused to eat and secretly threw away food. A psychiatrist was called in; the patient and her father were interviewed. She confessed that she had been told by her friends that she had put on weight, and as a result, she stopped eating on her own initiative. On questioning the patient, it emerged that she had good relationships with family and friends and had no history of trauma. During her psychiatric examination, she stated that she was obsessed with her body image, was afraid of gaining weight, and only ate lunch once a day. The patient was diagnosed with AN, and psychotherapy was started. The diet was started with 20 kcal/kg/day in the form of three main meals and three snacks, and the amount of calories were increased every day. Regular weight monitoring was carried out. During the three-week follow-up period in the hospital, the patient gained 2 kg. At the six-month follow-up, she reported eating three meals a day and not feeling tired, and the physical examination revealed that she had gained 19 kg (Figure 2). Amenorrhea was still present. Her BMI was reported as 18 kg/m^2^. After six months, the patient's hypercortisolemia markers were biochemically normal. The patient's laboratory results at six months are shown in Table 1.

## Discussion

In our case, we present hypercortisolemia that developed in a patient diagnosed with AN. Cortisol levels improved with the elimination of the underlying cause.

High nocturnal cortisol exposure in patients with CS increases the risk of metabolic and cardiovascular morbidity and mortality [9]. Changes in the cortisol rhythm and an increase in cortisol levels are observed in patients with AN [9,10]. Anorexia nervosa is a PCS that shares the clinical and biochemical features of CS [11,12]. Our case did not present with the typical stigmata of CS, such as proximal myopathy, purple striae, and easy bruising [9]. The clinical presentation of this case initially suggested an ectopic CS. The signs of hypercortisolemia were suggestive of ACTH-dependent CS. An ectopic focus was looked for as the MR of the pituitary gland was normal and the cortisol level was not suppressed by the 8 mg dexamethasone suppression test (DST) [9]. Examination with thoracic abdominal CT and Ga-68 scintigraphy did not reveal an ectopic focus.

Clinical and biochemical overlaps in PCS can complicate the differential diagnosis. In our case, the result of 2 mg DST did not lead to cortisol suppression, but the typical stigmata of CS were also absent. In a study of CS, AN, and healthy individuals, corticosteroid receptors on leukocytes were lower in patients with CS and AN than in healthy individuals [13]. Cortisol levels and corticosteroid receptors were negatively correlated in these two patient groups [14]. Chronic elevation of the HPA system in stress physiology is one of the pathophysiological mechanisms involved in hypercortisolemia [11-15]. Our patient also had concerns about her body shape and food resistance, and a psychotherapy and diet plan were created for this situation.

Apart from amenorrhea, our patient did not exhibit any features of CS. The study by Invitti et al. found that the receptor affinity of glucocorticoids decreased in AN patients compared to control subjects, and the metabolic substrates of glucocorticoids were reduced [16]. These results may explain the absence of Cushingoid features in patients with AN despite hypercortisolemia.

In patients with AN, we can find functional hypothalamic amenorrhea (FHA) associated with hypoleptinemia and underlying mental illness [17]. Functional hypothalamic amenorrhea is caused by impaired pulsatile secretion of the gonadotropic-releasing hormone [17]. After the condition of AN has improved, persistent amenorrhea is associated with persistent hypothalamic dysfunction [18]. In our case, although hypercortisolemia normalized due to the improvement in BMI, amenorrhea did not improve. This situation can be attributed to the fact that the patient has not yet reached the appropriate BMI.

Patients with AN exhibit GH resistance, i.e., they have high GH and low IGF-1 levels. This is associated with downregulation of GH receptor expression and low levels of GH-binding proteins [19]. The low T3 status in AN has been reported to be due to the inhibition of enzymatic deiodination of fT4 to metabolically active fT3 and elevated cortisol levels [20]. This condition, which resembles the sick euthyroid syndrome, is an adaptive mechanism that reduces energy expenditure at rest and contributes to energy conservation for vital functions, so no treatment is required [20]. We have encountered a similar situation in this case. Determinants of low bone density in patients with AN include hypogonadism, low BMI, low lean body mass, low IGF-1, and GH resistance [19,21]. We believe that bone density was not negatively affected in our case because the course of AN was short.

## Conclusions

Hypercortisolism results from conditions that can chronically activate the hypothalamic-pituitary-adrenal axis and occurs in many conditions, including psychiatric disorders such as AN. Our case presents a rare example of hypercortisolism with AN with a possible diagnosis of PCS. In patients with AN, CS should be suspected if typical symptoms and signs of CS are present. In PCS due to AN, the clinical and laboratory features are expected to improve with the necessary weight gain. Distinguishing between PCS and CS protects patients from unnecessary investigations and harmful treatments.
